# The impact of ^12^C^6^ heavy ion irradiation-induced cellular mutations on the replication of the foot-and-mouth disease virus and the role of* Cbr3*

**DOI:** 10.1007/s00018-025-05628-6

**Published:** 2025-06-28

**Authors:** Xiangdong Song, Shiyu Tao, Fanglan An, Xiaoming Li, Yanyan Chang, Xuerong Liu, Yan Cui

**Affiliations:** 1 College of Veterinary Medicine, Gansu Agricultural University, Lanzhou, China; 2 Vaccine Process Research Laboratory, China Agricultural Vet Biology and Technology Co., Ltd, Lanzhou, China

**Keywords:** ^12^C^6^ heavy ion, FMDV, *Cbr3*, PGE2, BHK-21

## Abstract

Foot-and-mouth disease (FMD) is an infectious disease in animals caused by the foot-and-mouth disease virus (FMDV). However, the mechanism of FMDV infection in host cells remains unclear. In this study, we utilized ^12^C^6^ heavy ion irradiation technology to process BHK-21 cells and systematically screened and evaluated cell lines with distinct virus replication characteristics. We subsequently employed proteomics to detect the differences between these cell lines and the control BHK-21 cell line following ^12^C^6^ heavy ion irradiation. Both cell lines exhibited common downregulation of cell adhesion molecules but also exhibited distinct upregulation pathways. In terms of immune and metabolic responses, BHK-5 infection triggered an immune response, including the upregulation of cytokine–cytokine receptor signaling pathways and lysosome-related pathways, while the upregulation of drug metabolism pathways enhanced the ability to metabolize exogenous substances. Conversely, BHK-7 infection tended to promote metabolic pathway changes that favor virus replication, such as the upregulation of folate biosynthesis, polysaccharide degradation, and linolenic acid metabolism pathways. Additionally, we observed significant downregulation of *Cbr3* in cell lines that promoted virus replication and significant upregulation in those that inhibited virus replication. Upon validating the results in *Cbr3* knockout cells, we found that knocking out *Cbr3* could increase FMDV replication by increasing the cellular content of prostaglandin E2 (PGE2), suggesting a close relationship between FMDV replication and PGE2 levels. This method can increase the production efficiency of FMDV vaccines while reducing manufacturing costs. This study innovatively employed ^12^C^6^ heavy ion irradiation technology to induce cell transformation and explored its impact on FMDV, offering a new perspective for understanding virus replication mechanisms and potentially providing a target and idea for developing novel antiviral strategies.

**Supplementary Information:**

The online version contains supplementary material available at 10.1007/s00018-025-05628-6.

## Introduction

Foot-and-mouth disease is an extremely contagious livestock disease that has caused considerable economic losses to the global animal husbandry industry. Foot-and-mouth disease virus (FMDV) spreads rapidly and has a complex replication mechanism, which poses major challenges for its prevention and control [1]. Although many studies have investigated the structure, transmission route, and immune response of FMDV, some mechanisms are still unclear [2]. Therefore, a comprehensive understanding of the replication mechanism of foot-and-mouth disease virus (FMDV) is extremely important.

In current basic research, the methods widely used in the study of the interaction between FMDV and the host include gene knockout [3], reverse genetics [4], research on virus gene function loss [5], the overexpression of specific genes [6], and bioinformatics analysis [7]. These methods play pivotal roles in antiviral research and disease prevention and control. However, the above methods have several shortcomings. For example, gene knockout construction is complex, reverse genetics technology is difficult, bioinformatics analysis results are uncertain, and there are limitations in viruses and hosts in terms of specific gene manipulation. In particular, when facing new diseases, finding relevant targets is often time-consuming and difficult.

As an advanced physical method, heavy ion irradiation technology has attracted much attention in the field of life sciences in recent years. Heavy ions possess unique physical and biological characteristics. Compared with traditional rays, they can deposit more concentrated and intense energy in organisms, thereby triggering specific biological changes in cells. It can induce a high mutation rate, a wide mutation spectrum, and excellent mutant stability [8]. The generated mutants can increase the possibility of discovering new virus-host interaction mechanisms. Moreover, this approach avoids the limitations that may be caused by purposeful manipulation of specific genes. It is closer to the variation in the natural state and can produce multiple different mutation types, providing richer materials for research [9–11]. These advantages make heavy ion irradiation a highly promising research tool. In addition, studies have shown that radiation can regulate cellular immune function and may affect virus replication in host cells [12–14]. For example, Sergey Iordansky's research revealed that X-ray IR has an activating effect on HIV-1 LTR-driven transcription and virus replication in major HIV-1 host cells [15]. Moreover, heavy ion irradiation has been proven to regulate immune function. Liu R et al. reported that after carbon ions induce Lewis cells, the expression of *TREX1* is significantly upregulated, whereas the expression of the immunogenic marker *IFN-β* is decreased [16]. Many early studies confirmed that interferon (*IFN)-β* has antiviral activity [17, 18]. Moreover, cells exposed to ionizing radiation can induce an immune response to a certain extent, involving multiple pathways, such as *TREX1*-exosomes and *STING* type I interferon [19]. Some of these pathways are closely related to virus infection and the infection mechanism of FMDV [20]. However, heavy ion irradiation mutagenesis also has shortcomings, which are specifically manifested as the randomness of mutations, making it difficult to obtain specific changes in the interaction between FMDV and the host in a targeted manner. Moreover, it may cause greater damage to cells and affect the normal physiological functions of cells.

In this study, we innovatively used heavy ion irradiation technology to induce BHK-21 cells. By evaluating the susceptibility to FMDV infection and the virus replication efficiency of many mutant cell lines generated, we screened the BHK-5 and BHK-7 cell lines, which inhibit and promote FMDV replication, respectively. Proteomic analysis of mutant BHK-5 and BHK-7 cells revealed that they have different response mechanisms than control cells after infection with FMDV. In addition, the important regulatory role of the key differential protein *Cbr3* in the FMDV replication process was determined. Further studies have shown that *Cbr3* affects FMDV replication by regulating the prostaglandin E2 (PGE2) degradation pathway, providing a new gene target for constructing a more comprehensive virus replication regulatory network. Finally, this study provides potential targets and innovative methods for the development of new antiviral strategies. In the future, heavy ion induction technology can be further used to study the interaction mechanism between viruses and host cells in detail, laying a foundation for the development of more effective antiviral drugs and vaccines.

## Materials and methods

### Cells and viruses

The wild-type BHK-21 cell line (ATCC CCL-10) was used in the cell culture experiments. The cells were maintained in DMEM (dulbecco's modified eagle medium,Gibco C11995500BT) supplemented with 5% fetal bovine serum (FBS; Gibco 10,099,141). The cultures were incubated in a humidified atmosphere containing 5% CO_2_ at 37 °C. Foot and mouth disease virus O/MYA98 strains were obtained from China Agricultural Vet Biology and Technology Co., Ltd.

### The induction of BHK-21 cells using ^12^C^6^ heavy ions

BHK-21 cells were cultured in a 35 mm cell culture dish at a density of 1 × 10^2^ cells/mL. They were induced by a carbon ion beam accelerated by the Lanzhou Heavy Ion Research Institute (HIRFL) of the Institute of Modern Physics, Chinese Academy of Sciences. The ion beam is (^12^C^6^), and the initial energy of the ion beam is 80.55 MeV/u. The cells were induced at doses of 5 Gy, 10 Gy, and 15 Gy. The control group was not induced by the ^12^C^6^ carbon ion beam. According to the heavy ion induction scheme in Table 1, the maximum dose rate, minimum dose rate threshold and optimal dose rate were set. There were 6 replicates in each dose group. After carbon ion beam irradiation, the medium was replaced with fresh DMEM containing 10% FBS. The cells were cultured in a humidified incubator at 37 °C and 5% CO₂ for 24 h to evaluate cell viability and determine cell mortality.

**Table 1:** ^12^C^6^-induced heavy ion doses (n = 6)

Sample	Optimal dosage (Gy)	Optimal dosage rate (Gy/min)	Minimum dose rate threshold (Gy/min)	Maximum dosage rate (Gy/min)
Cell-5 Gy	5.0	1.0	5.0	2.5
Cell-10 Gy	10	2.0	1.0	5.0
Cell-15 Gy	15	5.0	2.5	7.5
Control	0.0	0.0	0.0	0.0

### Screening monoclonal cells following irradiation with ^12^C^6^ heavy ions

After culturing for 48 h after irradiation with ^12^C^6^ heavy ions, stable cells were selected. The limited dilution method was used to adjust the cell concentration to 100 cells/15 mL. The resulting cell suspension was thoroughly mixed, and 150 μL of the mixture was transferred to each well of a 96-well cell culture plate. A 20 × microscope was used to confirm that each well contained a single clone. The cells were subsequently cultured in a humidified incubator at 37 °C with 5% CO_2_. When the cell confluence reached 90%, the single clone cells were transferred to a 6-well cell culture plate and continuously cultured for 10 generations. Compared with control BHK-21 cells, cells showing significant differences were identified as potential candidate cells.

### Evaluation of the replication capacity of FMDV in mutant cells following ^12^C^6^ heavy ion irradiation

Taking BHK-21 cells as the control group, 14 candidate mutant cell lines were cultured in a 6-well cell culture plate containing DMEM with 5% FBS to ensure that the number of cells in each well was consistent (the cell density of each experimental group was 3 × 10^5^ cells/ml). When the cells reached approximately 90% confluence, they were washed three times with PBS (phosphate-buffered saline, Solarbio, P1020-500). Then, the cells were incubated with FMDV (MOI = 1.0) in a humidified incubator at 37 °C and 5% CO_2_ for 1 h, washed three times with PBS, and 2 ml of DMEM containing 1% FBS was added. The uninfected cell group served as the control group. The cells were maintained in a humidified incubator at 37 °C and 5% CO_2_ for 16 h, after which the cultures and diseased cells were harvested. The samples were subsequently freeze-thawed three times at – 20 °C and stored overnight at – 20 °C. The 146S content of the FMDV antigen in each batch of samples was determined via sucrose density gradient centrifugation [21]. The absorbance of each fraction was measured at 259 nm via a continuous ultraviolet detector, and an absorption peak profile was generated. The 146S concentration in the samples was then calculated on the basis of the area under the absorption peak. Total RNA was extracted from the FMDV-infected cell culture medium via TRIzol reagent (Invitrogen, 15596026). The total RNA was subsequently reverse-transcribed into cDNA following the manufacturer’s instructions with the PrimeScript™ II First Strand Synthesis Kit (RR047A, Takara). The expression levels of the FMDV gene (FMDV-3D-F: GAACACATTCTTTACACCAGGAT, FMDV-3D-R: CATATCTTTGCCAATCAACATCAG) were measured alongside the internal reference gene GAPDH (GAPDH-F: ATGGCCTTCCGTGTTCCTAC, GAPDH-R: GCCTGCACCACCTTCTT). BHK-7, BHK-5, and control BHK-21 cell lines were trypsinized, counted via a Count STAR (RY074B2001, Countstar Rigel S2) cell counter, and diluted to a concentration of 2 × 10^3^ cells/mL before being transferred to T25 cell culture flasks. The experiment comprised 20 groups, each with 3 replicates. Cell counts were recorded every 4 h to determine the cell growth rate. BHK-5 and BHK-7 cells were passaged for 9 generations, with each generation labeled T1 (first generation), T2 (second generation), to T9 (ninth generation). Using BHK-21 cells as a control, the TCID_50_ and the concentration of intact virus particles (146S) were assessed every 3 generations. All the experiments were repeated at least three times, and relative mRNA expression levels were calculated via the threshold cycle (2^−△△*Ct*^) method.

### Quantitative proteomics analysis

In the uninfected group, BHK-7 and BHK-5 cells with excellent growth status and a viability of more than 98%, along with control BHK-21 cells, were selected and washed three times with PBS. In the infected group, the cells were infected with FMDV at an MOI of 0.1 for 6 h and then washed three times with PBS. Both groups were lysed in SDT (4% SDS, 100 mM Tris–HCl, pH = 7.6) buffer to extract proteins, which were subsequently quantified via a BCA protein assay kit. Trypsin digestion was carried out according to the FASP procedure. The digested peptides were desalted, concentrated, and reconstituted. Next, for SDS-PAGE, 20 µg of protein per sample was mixed with loading buffer, boiled, and separated on a gel. The protein bands were visualized by staining. Finally, LC–MS/MS analysis was performed on a timsTOF Pro mass spectrometer coupled to a NanoElute instrument with specific column setups and parameters. Additionally, LC–MS/MS analysis was conducted via a Q Exactive mass spectrometer coupled with an easy nLC system. The mass spectrometer was operated in positive ion mode, employing a data-dependent top20 method. The relevant parameters were set, and the instrument was run in peptide recognition mode.

### The *Cbr3*-knockout BHK-21 cell line was successfully established.

The *Cbr3* knockout BHK-21 cell line (BHK-21-KO-*Cbr3*) was generated via the CRISPR/Cas9 system. Complementary oligonucleotides encoding the gRNA (F: CACCGAGGCTGTGTATCGCGAGCC, R: *AAAC*GGCTCGCGATACACAGCCTC) were annealed and cloned and inserted into the BsmB I (Fermentas) site of the pSpCas9(BB)−2A-Puro (PX459) vector. According to the manufacturer's protocol, the pSpCas9(BB)−2A-Puro (PX459)-gRNA construct was transfected into BHK-21 cells via Lipofectamine™ 2000 Transfection Reagent (Thermo Fisher Scientific, 11668019). The cells were then treated with a specified concentration of puromycin (InvivoGen, ant-pr-1) for selection. The medium was replaced the following day, and the cells were passaged every other day. After 7 days, the cells were harvested for genomic DNA extraction and sequencing.

### Western blotting

The cells were lysed with lysis buffer containing phenylmethylsulfonyl fluoride (PMSF; Sigma, 11359061001) and subsequently clarified by centrifugation. Protein samples were resolved via precast 10% sodium dodecyl sulfate–polyacrylamide gel electrophoresis (SDS-PAGE), transferred onto polyvinylidene fluoride (PVDF) membrane, and blocked with 5% skim milk in TBST solution for 1 h. The membranes were incubated overnight at 4 °C with primary antibodies against *Cbr3* (K008652P, Solarbio, 1:1000), VP1 (Lanzhou Veterinary Research Institute, Chinese Academy of Agricultural Sciences, 1:1000), and β-actin (MA5-32540, Thermo, 1:20,000). The primary antibodies were then detected for 1 h using a goat anti-rabbit HRP-conjugated secondary antibody (31,460, Thermo, 1:10,000) and a goat anti-mouse HRP-conjugated secondary antibody (31430, Thermo, 1:10,000). The protein bands were visualized via an enhanced chemiluminescence detection reagent (Thermo) and imaged via Image Lab 4.1 software (Bio-Rad).

### Fluorescence microscopy

Wild-type BHK-21 (WT-BHK-21) and BHK-21-KO-*Cbr3* cells were infected with foot and mouth disease virus (FMDV) at a multiplicity of infection (MOI) of 1.0. After 8 h of infection, the cells were washed with PBS and fixed with 4% formaldehyde at room temperature for 10 min. After permeabilization, the cells were immunostained with anti-VP1 (Lanzhou Veterinary Research Institute, Chinese Academy of Agricultural Sciences, 1:50) and anti-β-actin (MA5-32540, Thermo, 1:1000) antibodies. Then, a goat anti-mouse IgG secondary antibody labeled with DyLight 649 fluorescence (A23610, Abbkine, 1:1000) and a goat anti-rabbit IgG secondary antibody labeled with DyLight 488 fluorescence (A23220, Abbkine, 1:1000) were used. The cell nuclei were stained with Hoechst 33258. Observation and imaging were performed under a confocal laser scanning microscope (LSM 900).

### Determination of TCID_50_

BHK-21 cells were seeded in 96-well plates at 90% confluence. Subsequently, in another culture plate, the virus samples (including BHK-7, BHK-5, control BHK-21, and BHK-21-KO-*Cbr3*) were serially diluted from 10⁻^1^ to 10⁻⁸. Then, 100 μL of the diluted sample was added to each well of cells and incubated in a humidified incubator at 37 °C and 5% CO₂ for 1 h. After that, the inoculum was removed, and DMEM containing 1% FBS was added to the cells. The cells were then continuously cultured in a humidified incubator at 37 °C and 5% CO₂ for 16 h. Finally, the TCID₅₀ value was determined via the Reed-Muench method.

### Quantification of PGE2 levels

BHK-21 cells and BHK-21-KO-*Cbr3* cells were cultured in a 6-well cell culture plate. Upon reaching 90% confluence, the cells were trypsinized and subsequently washed three times with PBS. Both cell types were then infected with foot-and-mouth disease virus (FMDV) at a multiplicity of infection (MOI) of 0.1 for 6 h, followed by three additional washes with PBS. A total of 1 × 10^6^ cells from each sample were subjected to five freeze–thaw cycles and centrifuged at 600 × g, and the resulting supernatant was collected according to the manufacturer's instructions (Hamster Prostaglandin E2 (PGE2) ELISA Kit, MLBio, Catalog, ml003209). The PGE2 content was subsequently measured (Appendix 1).

### Statistical analysis

The experimental data were analyzed through Student’s two-tailed unpaired *t*-tests and two-way analysis of variance (ANOVA) with the aid of GraphPad Prism 9. *P-*values of < 0.05 were statistically significant.

## Results

### The impact of ^12^C^6^ heavy ion irradiation on the replication of foot-and-mouth disease virus

**Fig. 1:**
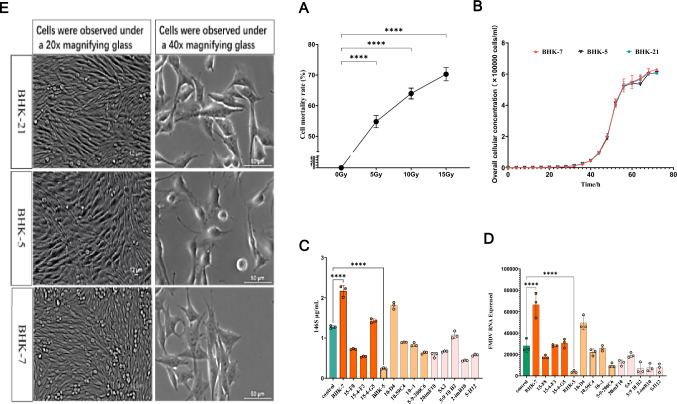
**A** BHK-21 cells were induced with ^12^C^6^ heavy ions at doses of 5 Gy, 10 Gy, and 15 Gy. Cell viability was assessed via trypan blue staining and detected via a Count STAR (Countstar Rigel S2, RY074B2001), and the mortality rate was calculated. The data represent the means and standard errors from six independent experiments. **B** Changes in cell density over time (0 to 72 h) were monitored for control (BHK-21) cells and mutant BHK-7 and BHK-5 cells via a Count STAR. All the cells to be detected had the same initial density. Growth curves were plotted on the basis of the mean ± standard error from three independent experiments. **C** Fourteen mutant cell lines and control (BHK-21) cells were adjusted to the same initial density and infected with FMDV at an MOI of 1.0 for 16 h. The content of FMDV antigen 146 s was quantified. The results are presented as the means and standard errors from three independent experiments. **D** The expression level of the FMDV 3D gene in 14 mutant cell lines and control BHK-21 cells infected with FMDV (MOI = 1.0) for 16 h was measured by qRT-PCR with GAPDH as the reference. Relative expression levels were calculated via the 2^−ΔΔCt^ method. The data represent the means and standard errors from three independent experiments. **E** An inverted microscope was used to capture the morphological characteristics of fully fused control BHK-21, BHK-7, and BHK-5 cells under bright-field conditions. The magnification was achieved by combining the appropriate eyepieces and a 20 × objective lens. For partially fused cells, magnification was obtained by combining the same eyepieces and a 40 × objective lens under the same bright-field conditions.. The data are presented as the means ± SDs from three independent experiments and were analyzed via Student's two-tailed unpaired *t*-tests. *, *P* < 0.05; **, *P* < 0.01; ***, *P* < 0.001; ****, *P* < 0.0001

Wild-type BHK-21 cells were exposed to ^12^C^6^ heavy ions at doses of 5 Gy, 10 Gy, and 15 Gy. The impact of these three doses on cell viability was assessed, revealing a significant decrease in cell survival rates (Fig. 1A). Subsequently, surviving cells with stable morphology postirradiation were subjected to limiting dilution to generate monoclonal cell lines, which were then continuously passaged and screened. Throughout this process, the majority of cells exhibited reduced growth and underwent apoptosis due to ^12^C^6^ heavy ion exposure, while only a small fraction thrived. Ultimately, 14 stable monoclonal cell lines were established. Specifically, five cell lines originated from the 15 Gy dose group, designated BHK-7, 10-1, 15-4-f3, 15-4-g3, and 10-D4 mutants; five cell lines emerged from the 10 Gy dose group, identified as 10.59 C4, 15-f 8, 5–9–200 C6, 5A2, and 20imF10 mutants; and four cell lines arose from the 5 Gy dose group, named the 5:9 10 B2, 2-imB10, 5-h12, and BHK-5 mutants. After the infection of 14 stable mutant cell lines with FMDV, the levels of complete FMDV particles (146S) and FMDV gene expression were subsequently analyzed (Fig. 1C). Both the mutant cells and the control BHK-21 cells were infected with FMDV (MOI = 1.0) for 16 h, maintaining a consistent cell density. The results revealed that the 146S content in 4 mutant cell lines from the 5 Gy group and 5 mutant cell lines from the 10 Gy dose group was significantly lower than that in the control BHK-21 cells. Notably, the 146S content in BHK-5 cells was markedly reduced to 0.24067 ng/ml, representing an 81.07% decrease compared with that in BHK-21 cells (*p* < 0.0001). These findings suggest that, compared with control BHK-21 cells, mutant BHK-5 cells may have a potential inhibitory effect on FMDV. Conversely, when the mutant BHK-7, 15-f8, and 15-4-g5 cells from the 15 Gy dose group were infected with FMDV, their 146S content exceeded that of the control BHK-21 cells. Specifically, the 146S concentration in mutant BHK-7 cells increased to approximately 2.171 ng/ml, a 70.67% increase over that in control BHK-21 cells. This finding indicates that, under the same conditions, the mutant BHK-7 cells enhance FMDV replication to a significantly greater extent than the control BHK-21 cells do. The FMDV gene expression levels after infection were measured via RT-qPCR (Fig. 1D). The FMDV gene copy number was highest in the 15 Gy dose group, followed by the 10 Gy and 5 Gy dose groups, which was in line with the 146S detection results.

Furthermore, the growth curves of the mutant BHK-5 and BHK-7 cells were compared with those of the control cells (Fig. 1B). The growth rates of the strains were essentially identical, thus ruling out any potential confounding effects of differential cell growth rates on viral replication in infected cells. Microscopic examination of the morphology of the fused and unfused cells (Fig. 1E) revealed that postfusion BHK-5 cells exhibited a spindle shape with a flattened center, whereas the morphology of BHK-7 and control BHK-21 cells remained similar. Unfused BHK-5 cells displayed a more prominent central region, whereas the morphology of BHK-21 and BHK-7 cells was similar but with minor variations.

### Assessment of BHK-5 and BHK-7 in FMDV replication.

**Fig. 2:**
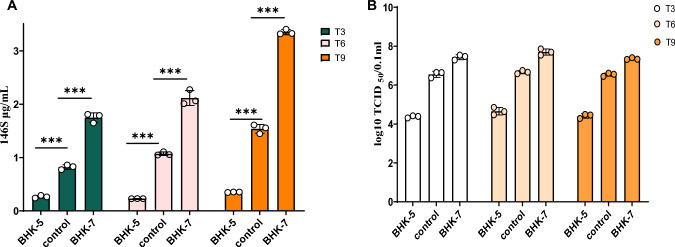
**A** Control BHK-21 cells and mutant BHK-7 and BHK-5 cells were passaged 9 times and infected with FMDV (MOI = 1.0) for 16 h every 3 passages to monitor changes in the content of the FMDV antigen 146S. **B** Control BHK-21 cells and mutant BHK-7 and BHK-5 cells were passaged 9 times and infected with FMDV (MOI = 1.0) for 16 h every 3 passages to assess changes in TCID50 values. The data represent the means ± standard deviations from three independent measurements. The data are presented as the means ± SDs from three independent experiments and were analyzed via Student's two-tailed unpaired *t*-tests. *, *P* < 0.05; **, *P* < 0.01; ***, *P* < 0.001; ****,* P* < 0.0001

To assess the stability of the mutant cell lines BHK-5 and BHK-7, they were continuously passaged for nine generations. In every three passages, the cells were infected with FMDV (MOI = 1.0) for 16 h, and the levels of 146S and FMDV replication were monitored. The results indicated that the cell lines maintained consistent viral replication capabilities throughout the passage process. In summary, the screening process successfully identified BHK-7 cells as a cell line that promotes foot-and-mouth disease virus replication and BHK-5 cells as a cell line that inhibits replication (Fig. 2A, B).

### Proteomic analysis of mutant BHK-7, BHK-5, and control BHK-21 cells before and after FMDV infection

A label-free 4D quantitative proteomics approach was employed to examine two groups: the FMDV-infected group and the non-FMDV-infected group. In the FMDV-infected group, BHK-21 cells infected with foot-and-mouth disease virus (FMDV) served as the control, whereas BHK-5 and BHK-7 cells infected with FMDV composed the experimental group of cells. In the control group, BHK-21 cells were the control, and BHK-5 and BHK-7 cells were the experimental group of cells.

For significant difference analysis, the fold change (FC) and P value of protein expression differences between BHK-5 and control BHK-21, as well as between BHK-7 and control BHK-21, in the FMDV-infected and non-FMDV-infected groups were calculated. The screening criteria were FC > 2 (upregulation greater than 2 times or downregulation less than 0.50 times) and a *P*-value < 0.05 to determine the number of upregulated and downregulated proteins among the groups.

**Fig. 3:**
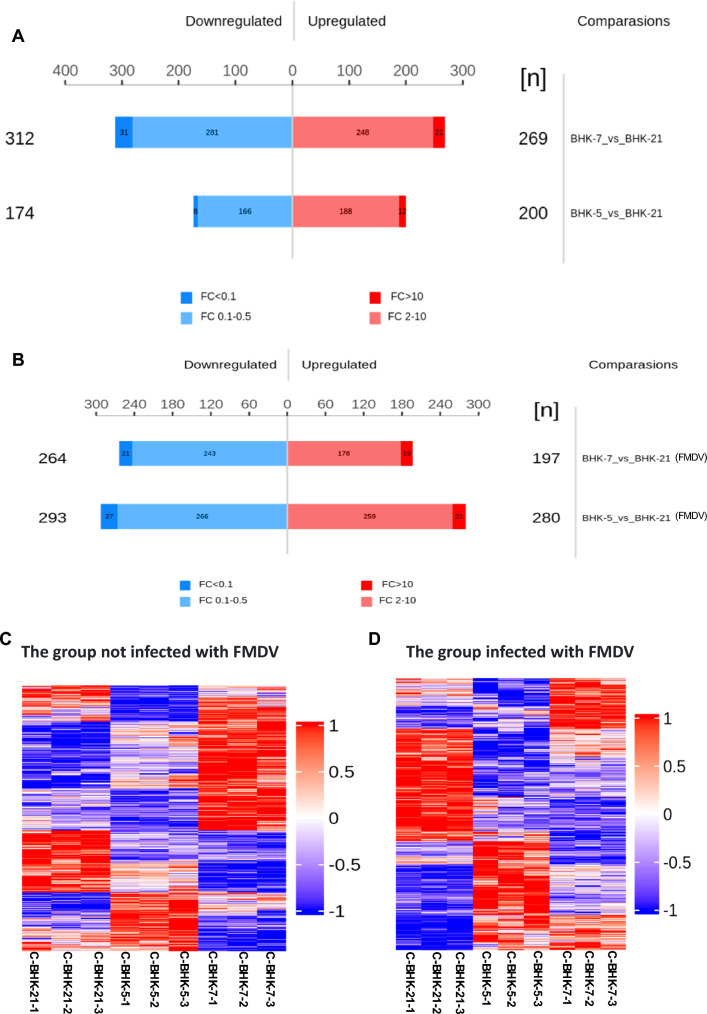
**A** Statistical graph of significantly different proteins between BHK-5, BHK-7, and the control (BHK-21); **B** Statistical graph of significantly different proteins after FMDV infection in BHK-5, BHK-7, and the control (BHK-21); **C** and **D** Heatmaps of significantly different protein expression before and after FMDV infection in BHK-5, BHK-7, and the control (BHK-21)

Compared with control BHK-21, a total of 374 differentially expressed genes (DEGs) were identified in BHK-5, with 174 DEGs significantly downregulated and 200 DEGs significantly upregulated. In BHK-7, a total of 581 DEGs were identified, of which 312 DEGs were significantly downregulated, and 269 DEGs were significantly upregulated (Fig. 3A). Compared with control BHK-21, a total of 573 DEGs were identified in BHK-5 cells after FMDV infection, with 293 DEGs significantly downregulated and 280 DEGs significantly upregulated. In BHK-7, a total of 461 gene fragments were identified, with 264 gene fragments significantly downregulated and 197 gene fragments significantly upregulated (Fig. 3B). We subsequently plotted the overall changes in protein expression of control BHK-21 and mutant BHK-5 and BHK-7 cells before and after FMDV infection (Fig. 3C, D). The results demonstrated that there were significant differences in protein expression between control BHK-21 cells and mutant BHK-5 and BHK-7 cells.

**Fig. 4:**
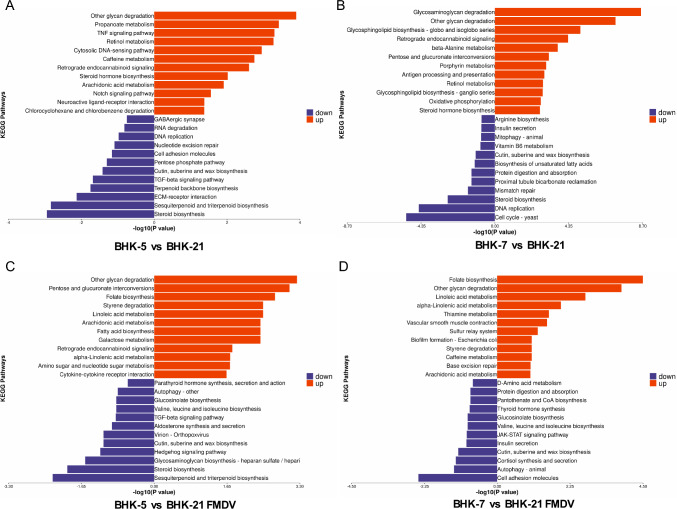
**A** KEGG pathway map associated with proteins differentially expressed between BHK-5 and BHK-21 cells. **B** KEGG pathway map associated with proteins differentially expressed between BHK-7 and BHK-21 cells. **C** KEGG pathway map associated with proteins differentially expressed between BHK-5 and BHK-21 cells post-FMDV infection. **D** KEGG pathway map associated with proteins differentially expressed between BHK-7 and BHK-21 cells post-FMDV infection. The x-axis represents the -log10(*p*-value) derived from Fisher's exact test, indicating the statistical significance of pathway enrichment. The y-axis lists the names of the pathways. The upregulated and downregulated pathways are denoted by red (right) and blue (left) bars, respectively

To elucidate the comprehensive metabolic pathway enrichment characteristics of all the differentially expressed proteins. Following the annotation steps, the studied proteins were blasted against the online Kyoto Encyclopedia of Genes and Genomes (KEGG) database (http://geneontology.org/) to retrieve their KEGG orthology identifications and subsequently mapped to pathways in KEGG. Enrichment analyses were performed via Fisher's exact test, considering all the quantified proteins as background datasets. Benjamini–Hochberg correction for multiple testing was further applied to adjust the derived p values. Only functional categories and pathways with *p* values under a threshold of 0.05 were considered significant. BHK-5 and BHK-7, induced by heavy ions, exhibited significant KEGG pathway alterations and intergroup proteomic differences before and after infection with foot-and-mouth disease virus (FMDV). In BHK-5, compared with those in the control BHK-21 without FMDV infection, the downregulated pathways involved primarily steroid biosynthesis, sesquiterpene and triterpene biosynthesis, and complement and coagulation cascades; the upregulated pathways involved mainly drug metabolism-related pathways (e.g., drug metabolism-other enzymes, cytochrome P450 for metabolizing xenobiotics) and arachidonic acid metabolism. Post-FMDV infection, cell adhesion molecules, the p53 signaling pathway, and autophagy-related pathways were significantly downregulated. The downregulation of cell adhesion molecules could impact intercellular connections and communication, changes in autophagy regulation were observed, and the downregulation of the p53 signaling pathway might influence cell apoptosis and DNA damage repair. Concurrently, metabolism-related pathways (drug metabolism-other enzymes, drug metabolism-cytochrome P450, and arachidonic acid) remained prominent, while new upregulated pathways, such as alcoholic liver disease and complement and coagulation cascades, emerged (Fig. 4A, C).

When BHK-7 cells were not infected with FMDV, compared with those in control BHK-21 cells, the downregulated pathways included primarily cell cycle-related pathways (e.g., the cell cycle in yeast and the cell cycle), steroid biosynthesis, and ECM-receptor interactions, among others. The upregulated pathways included mainly the lysosome, glycosaminoglycan degradation, and retrograde endocannabinoid signaling pathways, among others. Upon infection with FMDV, ECM-receptor interactions, cell adhesion molecules, and the PI3K-Akt signaling pathway were significantly downregulated. Among the upregulated pathways, new pathways, such as folate biosynthesis, motor proteins, and lysosomes, emerged (Fig. 4B, D).

**Fig. 5:**
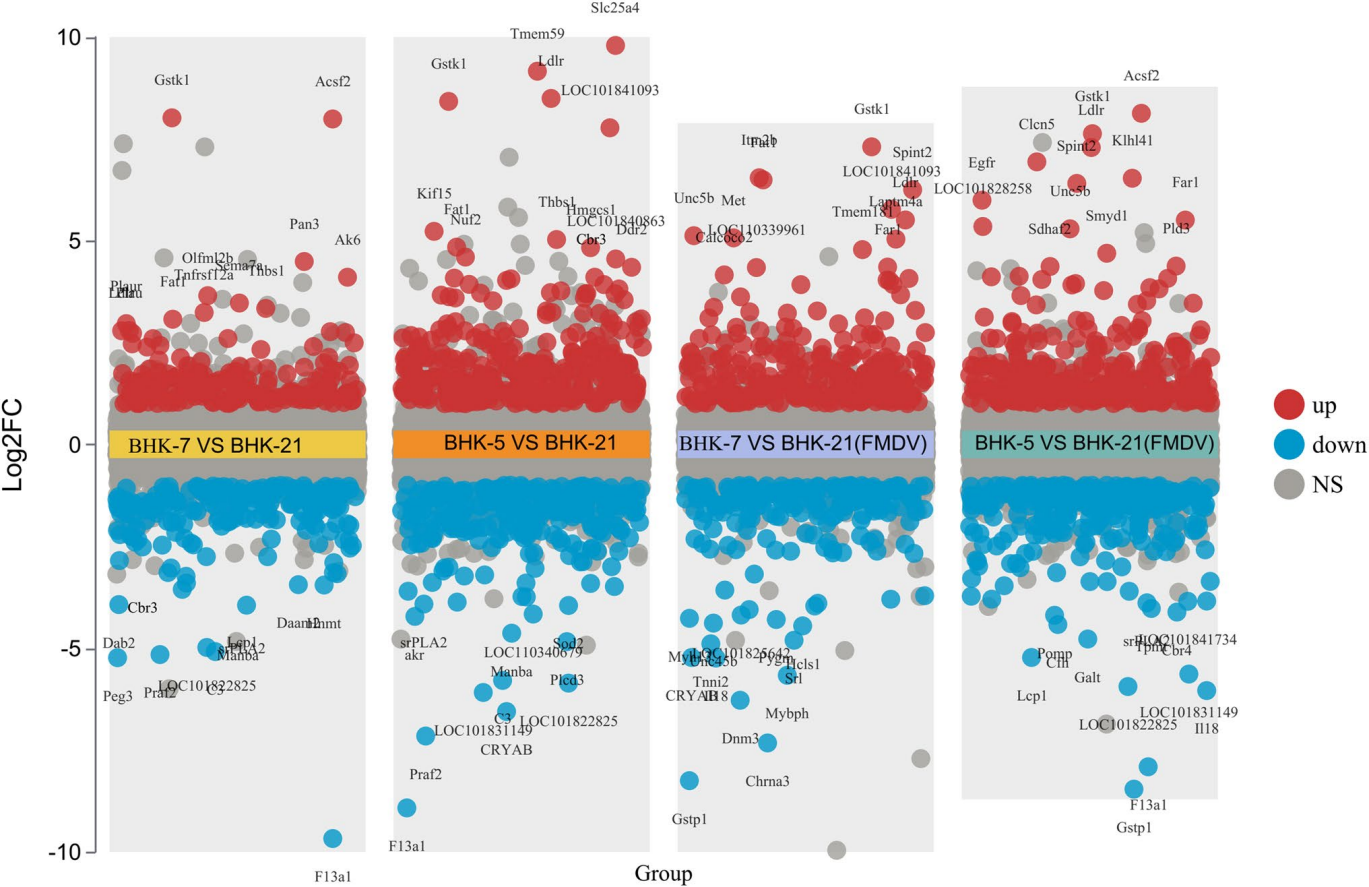
The multi-group differential volcano plots generated on the basis of the log fold change (logFC) and* p*-value of differentially expressed proteins (DEPs) in BHK-5, BHK-7, and control BHK-21 cells. The top 10 proteins from each group are labeled. The red dots indicate upregulated DEPs, the green dots denote downregulated DEGs, and the gray dots represent genes whose expression did not significantly change

For the proteomic DEGs, we subsequently constructed a volcano plot of the DEGs via two criteria, the fold change and* p*-value, as shown in Fig. 5. The analysis revealed that *GSTK1, TMEM59, LDIR, EGFR, F13A1,* and *LOC101822825* were significantly different. Notably, *Cbr3* significantly differed in its upregulation and downregulation patterns between the groups (differential volcano maps are marked with green circles). Through KEGG enrichment analysis, *Cbr3* was found to be enriched mainly in the arachidonic acid metabolism pathway, and this pathway was significant in the proteomic enrichment analysis. We subsequently analyzed the expression levels of the main enriched proteins in this KEGG pathway in BHK-5 and BHK-7 cells and control BHK-21 cells and found that *Cbr3* in this pathway was significantly different (Appendices 1, 2). Therefore, *Cbr3* may play a certain role in FMDV replication. To verify this hypothesis, we conducted further verification experiments.

The carbonyl reductase *Cbr3* catalyzes the reduction of many carbonyl compounds with biological and pharmacological activities to the corresponding alcohols. This enzyme is classified as an NADPH-dependent monomeric oxidoreductase [22]. *Cbr3* consists of three exons with a length of 11.2 kb and is closely related to another carbonyl reductase gene, *Cbr1*. Prostaglandin E2 (PGE2) plays a crucial role in immune and inflammatory responses. Elevated PGE2 levels are usually associated with intensified inflammatory responses and immunosuppression. Enrichment analysis revealed that *Cbr3* participates in the degradation of PGE2 through the arachidonic acid metabolism pathway [23]. In BHK-5 virus-suppressing cells, an increase in *Cbr3* expression may reduce PGE2 levels, thereby alleviating inflammatory responses and immunosuppression and enhancing the antiviral ability of cells. Conversely, in BHK-7 virus-promoted replication cells, a decrease in *Cbr3* expression or inhibition of its activity may lead to an increase in PGE2 levels, thereby intensifying inflammatory responses and immunosuppression and providing a favorable environment for virus replication.

### Establishment of a *Cbr3* knockout BHK-21 cell line

**Fig. 6:**
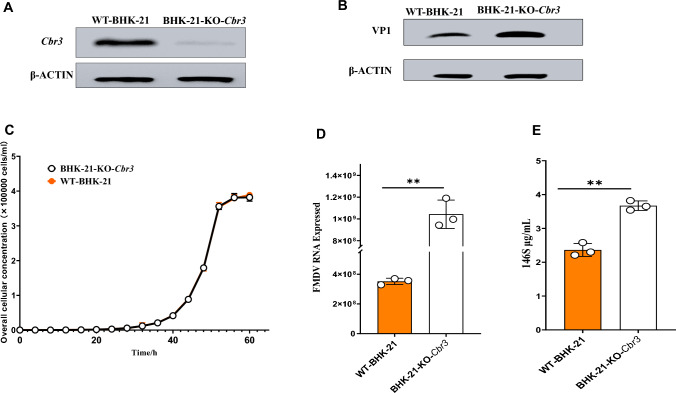
**A** *Cbr3* expression was detected via Western blotting via an anti-*Cbr3* antibody, with β-actin serving as the loading control to ensure uniform sample loading. **B** WT-BHK-21 and BHK-21-KO-*Cbr3* cells were infected with FMDV (MOI = 1.0). At 16 h postinfection, the protein levels of FMDV VP1 were assessed via Western blotting, with β-actin used as the internal reference. **C** WT-BHK-21 and BHK-21-KO-*Cbr3* cells were seeded in T25 flasks at a density of 2.0 × 10^3^ cells/mL. Trypsinized cells were counted every 4 h to construct growth curves. D: WT-BHK-21 and BHK-21-KO-*Cbr3* cells were seeded at a density of 3.0 × 10^5^ cells/mL and infected with FMDV (MOI = 1.0) for 16 h. The relative expression of FMDV 3D mRNA was quantified via qRT-PCR. All experiments were performed in triplicate, and the data are presented as the means ± SDs (n = 3). E: WT-BHK-21 and BHK-21-KO-*Cbr3* cells were infected with FMDV (MOI = 1.0) at a density of 3.0 × 10^5^ cells/mL for 16 h. The content of FMDV antigen 146 s was measured. The data are presented as the means ± SDs from three independent experiments and were analyzed via Student's two-tailed unpaired* t*-tests. *, *P* < 0.05; **, *P* < 0.01; ***, *P* < 0.001; ****, *P* < 0.0001

To investigate the impact of *Cbr3* knockout on FMDV replication, a BHK-21 cell line with *Cbr3* knockout, designated BHK-21-KO-*Cbr3*, was constructed via the CRISPR/Cas9 system. The detailed procedures were as follows. An sgRNA expression plasmid was constructed and transfected into BHK-21 cells via Lipofectamine 2000. Puromycin selection was subsequently applied to screen for successfully transfected cells. PCR primers were designed to amplify regions 100 bp upstream and downstream of the sgRNA target site. DNA was extracted from the cells, and amplification and sequencing were performed (see supplementary materials for verification results). Compared with WT-BHK-21 cells, deletions were observed near the *Cbr3* target site. Western blot analysis further confirmed that *Cbr3* protein expression was nearly completely abolished in the knockout cell line (Fig. 6A). To assess the potential effects of *Cbr3* knockout on cell growth, growth curves of the knockout cell line and WT-BHK-21 cells were compared. The results indicated that the growth patterns of both cell lines were essentially identical (Fig. 6C), suggesting that *Cbr3* knockout does not impair cell proliferation. In summary, the *Cbr3* knockout cell line was successfully established.

### Knockout of *Cbr3* increased FMDV replication.

**Fig. 7:**
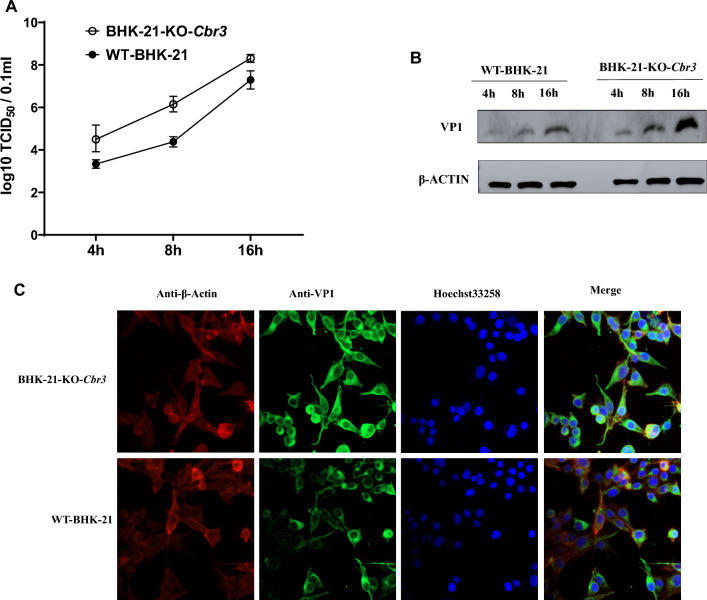
**A** WT-BHK-21 and BHK-21-KO-*Cbr3* cells were infected with FMDV (MOI = 1.0). At 4, 8, and 16 h postinfection, the protein levels of FMDV VP1 were assessed via Western blotting, with β-actin used as the internal reference. B: TCID₅₀ values were determined by infecting WT-BHK-21 and BHK-21-KO-*Cbr3* cells with FMDV. The experiments were conducted three times, and the data are expressed as the means ± SDs (n = 3). C: WT-BHK-21 and BHK-21-KO-*Cbr3* cells were infected with FMDV (MOI = 1.0) for 8 h. Immunofluorescence staining followed by confocal microscopy was used to observe the signal of the FMDV protein VP1. The green signal represents VP1, the nucleus was stained with Hoechst 33,258, and the red signal represents β-actin. The data are presented as the means ± SDs from three independent experiments and were analyzed via Student's two-tailed unpaired *t-*tests. *, *P* < 0.05; **, *P* < 0.01; ***, *P* < 0.001; ****, *P* < 0.0001

To evaluate the replication efficiency of foot-and-mouth disease virus (FMDV) in wild-type BHK-21 cells (WT-BHK-21) and *Cbr3* gene knockout BHK-21 cells (BHK-21-ko-*Cbr3*), the cells were infected with a multiplicity of infection (MOI) of 0.1 for 16 h. Total RNA was subsequently extracted from the cells and reverse transcribed to quantify the mRNA expression level of FMDV. Compared with that in WT-BHK-21 cells, the replication of FMDV in BHK-21-KO-*Cbr3* cells was significantly greater (Fig. 6D), and the content of FMDV antigen 146 also significantly increased, as expected (Fig. 6E). In addition, the virus titers of BHK-21-ko-*Cbr3* and WT-BHK-21 cells at 4, 8, and 16 h were measured and compared (Fig. 7A, B). Western blotting detection of FMDV VP1 also revealed that the expression of BHK-21-KO-*Cbr3* infected with FMDV was greater than that of WT-BHK-21 at different time points. Finally, the replication of FMDV in these two cell types after 8 h of BHK-21-KO-*Cbr3* and WT-BHK-21 infection with FMDV was visualized via confocal laser microscopy (Fig. 7C). These data indicate that knockout of the *Cbr3* gene significantly enhances the replication of FMDV in BHK-21 cells.

### *Cbr3* influences FMDV replication through the regulation of PGE2 degradation

**Fig. 8:**
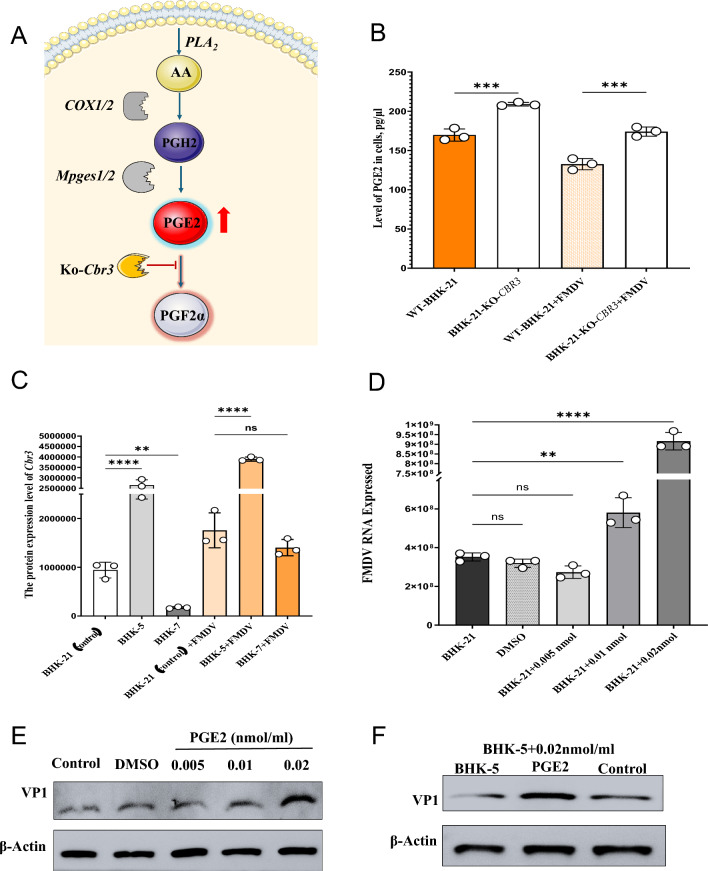
**A** represents the synthesis pathway of prostaglandin E2 (PGE2) in cells (*AA* arachidonic acid; *PGH2* prostaglandin H2; *PGE2* prostaglandin E2; *PGF2α* prostaglandin F2α). **B** PGE2 content in cells was determined via a PGE2 ELISA kit. The experiment included three replicates, and the results are expressed as the mean ± standard deviation (SD) (n = 3). The protein expression level of *Cbr3* in BHK-21-ko-*Cbr3* and control BHK-21 cells was evaluated. D. Wild-type BHK-21 cells were cultured in 6-well plates and treated with 0.005, 0.01, or 0.02 nmol/ml PGE2. The cells were infected with the foot-and-mouth disease virus (FMDV) for 16 h at a multiplicity of infection (MOI) of 1.0. The relative expression level of FMDV 3D mRNA was detected via real-time quantitative PCR (qRT-PCR). The experiment included three replicates, and the results are expressed as the means ± SDs (n = 3). When exogenous PGE2 was added to BHK-5 cells, the protein expression of *Cbr3* was significantly greater than that in control BHK-21 cells. The cells were infected with FMDV (MOI = 1.0) for 16 h. The expression of the FMDV VP1 protein was detected by Western blotting, with β-actin used as the loading control. F. BHK-5 cells were cultured in 6-well plates, 0.02 nmol/ml PGE2 was added, and the cells were infected with FMDV (MOI = 1.0) for 16 h. The expression of the FMDV VP1 protein was detected by Western blotting, with β-actin serving as the internal reference. The data are presented as the means ± SDs from three independent experiments and were analyzed via Student's two-tailed unpaired *t*-tests. *, *P* < 0.05; **, *P* < 0.01; ***, *P* < 0.001; ****, *P* < 0.0001

PGE2 is an eicosanoid lipid mediator with diverse functions, such as promoting cell proliferation and stem cell expansion and exerting immunosuppressive effects. Omics analysis revealed that the *Cbr3* protein plays a role in the degradation of PGE2 within the arachidonic acid metabolism pathway (Fig. 8A). We measured the levels of PGE2 in both BHK-21-KO-*Cbr3* cells and WT-BHK-21 cells. These findings indicate that *Cbr3* knockout leads to increased intracellular PGE2 accumulation and reduced degradation of PGE2 to PGF2α (Fig. 8B). To investigate whether PGE2 influences FMDV replication, we added 0.005 nmol, 0.01 nmol, or 0.02 nmol of PGE2 to WT-BHK-21 cells and assessed their condition 16 h post-FMDV infection. The results demonstrated that as the concentration of added PGE2 increased, so did the replication rate of FMDV in WT-BHK-21 cells (Fig. 8D, E). In BHK-5 cells, which were previously selected for their ability to inhibit viral replication, the expression level of the *Cbr3* protein was notably greater than that in control BHK-21 cells and the virus-promoting cell line BHK-7. These findings suggest that overexpression of the *Cbr3* protein might facilitate the degradation of PGE2. To confirm this hypothesis, we supplemented BHK-5 cells with 0.02 nmol of PGE2, and the results aligned with our expectations: the addition of PGE2 led to a significant increase in FMDV replication in BHK-5 cells (Fig. 8C, F). Collectively, these findings suggest that the *Cbr3* protein modulates FMDV replication by regulating cellular PGE2 levels.

## Discussion

This study innovatively employed ^12^C^6^ heavy ion irradiation technology to induce BHK-21 cells, demonstrating numerous significant advantages. ^12^C^6^ Heavy ions can deposit more concentrated and intense energy within organisms, enabling the precise induction of specific genetic and epigenetic alterations in cells. Compared with conventional mutagenesis techniques, heavy ions offer superior control over the extent and scope of mutagenesis. This precision significantly enhances the efficiency of screening cell lines with targeted biological traits while ensuring the accuracy and reliability of the screening outcomes. Furthermore, ^12^C^6^ heavy ion screening can produce a substantial number of mutant cell lines in a relatively brief timeframe, providing ample resources for in-depth investigations into the replication mechanisms of foot-and-mouth disease virus (FMDV). Research has indicated that varying doses of ^12^C^6^ carbon ions can induce gene mutations, modulate cellular immune responses, alter cellular immune parameters, and impact the expression of interferons and cytokines [24–26]. However, there is a lack of literature reporting on the interactions between heavy ion-induced irradiated cells and viruses.

This study successfully generated a series of mutant cells by irradiating wild-type BHK-21 cells with varying doses of ^12^C^6^ heavy ions. This finding underscores the critical role of host cells in the replication of foot-and-mouth disease virus (FMDV). As the environment for viral survival and replication, any alteration in the characteristics of host cells inevitably impacts viral replication. By utilizing heavy ion irradiation technology, we can intentionally modify the characteristics of host cells, enabling more in-depth investigations into the interactions between viruses and host cells. The results showed that a lower dose (5–10 Gy) of ^12^C^6^ heavy ion irradiation inhibited FMDV replication, whereas a higher dose (15 Gy) promoted viral proliferation. This trend aligns with the findings of Lumniczky K [26], suggesting that the impact of ^12^C^6^ heavy ion irradiation on host cells varies at different doses, leading to distinct effects on viral replication. At lower doses, irradiation has an immunomodulatory effect, potentially enhancing the immune response of host cells and thereby inhibiting viral replication; at higher doses, it primarily exerts an immunosuppressive effect, possibly creating a more favorable environment for viral replication. Through screening and evaluation, we discovered that the mutant somatic cell line BHK-5 significantly inhibited the replication of the FMDV virus, whereas BHK-7 markedly promoted FMDV replication.

To gain a deeper understanding of the differences between these two types of mutant somatic cells and control cells, we performed proteomic analysis. The results indicated that both cell types exhibited a common downregulation of cell adhesion molecule pathways postinfection, yet they also displayed distinct upregulated pathways, demonstrating that they have different response mechanisms to viral infection. In terms of immunity and metabolism, BHK-5 initiates an immune response following infection, including the upregulation of cytokine-cytokine receptor signaling pathways and related pathways such as lysosomes. Moreover, the upregulation of drug metabolism-related pathways enhances the metabolic capacity for exogenous substances. These findings suggest that upon viral infection, BHK-5 cells initiate an immune response and increase their metabolic capacity to resist the virus [20, 27]. Heavy ion irradiation may further potentiate the activation of these immune and metabolic pathways in BHK-5 cells, thereby enhancing their ability to combat the virus. Conversely, BHK-7 cells tend to promote metabolic pathway alterations that facilitate viral replication, such as folate biosynthesis and glycan degradation pathways. The upregulation of pathways, such as linoleic acid metabolism, may be associated with increased energy demands following cellular infection [28, 29], implying that upon viral infection, BHK-7 cells create a favorable metabolic environment for viral replication. Heavy ion irradiation may exacerbate these metabolic pathway changes, thus promoting viral proliferation. Further investigations are needed to elucidate the specific mechanisms involved.

Cytokines, a class of polypeptide signaling molecules, regulate various biological processes by binding to cell surface receptors, including *IL-6, TNFα, MCP-1, IL-2,* and *IL-4* [30]. In response to viral and microbial infections, host cells defend themselves by releasing cytokines. Research has demonstrated that foot-and-mouth disease virus (FMDV) infection can induce cytokine production [31, 32], indicating that host cells employ multiple pathways to combat viral infections. Prostaglandin E2 (PGE2) is a significant immunomodulatory factor; different prostaglandins (PGE2, PGI2, PGD2, PGF2α) contribute to the inflammatory process. Viral infection activates the immune system, leading to the release of inflammatory mediators such as proinflammatory cytokines (*IL-6, IL-1β*, and *TNF-α*) and eicosanoids (PG and LT) [33]. Additionally, PGE2 facilitates the replication of viruses such as cytomegalovirus, herpes simplex virus (HSV), and coxsackievirus B2 by interacting with viral transcription and translation [34]. PGE2 is synthesized from arachidonic acid via the action of cyclooxygenase (*COX*) [35]. However, the impact of PGE2 downstream degradation on viral activity remains unexplored.

In this study, the *Cbr3* protein was markedly downregulated in the BHK-7 cell line, which facilitates FMDV replication, whereas it was markedly upregulated in the BHK-5 cell line, which hinders FMDV replication. These findings suggest that the *Cbr3* protein exerts a significant regulatory influence on the FMDV replication process. Previous omics enrichment analyses revealed that the *Cbr3* protein modulates the degradation of prostaglandin E2 (PGE2) within the arachidonic acid pathway. In BHK-5 cells, which inhibit viral replication, increased expression of *Cbr3* may lead to a reduction in PGE2 levels, thus mitigating the inflammatory response and immunosuppression and consequently bolstering the antiviral capabilities of these cells. Conversely, in BHK-7 cells, which promote viral replication, low expression or inhibited activity of *Cbr3* could result in elevated PGE2 levels, thereby exacerbating the inflammatory response and immunosuppression and providing a conducive environment for viral replication. To validate this hypothesis, we conducted additional research using the *Cbr3* knockout BHK-21 cell line. These findings confirmed the critical role of the *Cbr3* gene in suppressing FMDV replication. Upon *Cbr3* knockout, the replication rate of FMDV markedly increased, as did the concentration of intact FMDV virions (146S). These results suggest that the presence of the *Cbr3* gene is essential for inhibiting FMDV replication. Furthermore, by comparing the growth curves of the knockout cell line with those of the wild-type cell line, we observed that the *Cbr3* gene knockout did not impact cell growth. This observation provides a valuable reference for further investigations into the function of the *Cbr3* gene and suggests that controlling FMDV replication through the regulation of *Cbr3* gene expression is feasible without compromising cell viability.

This discovery complements other virus replication-related genes identified by previous researchers, offering new insights into the construction of a more comprehensive virus replication regulatory network. Moreover, this study identified potential targets and innovative approaches for developing novel antiviral strategies. Future research could further explore the use of ^12^C^6^ heavy-ion-induced technology to screen for additional cell lines with specific biological traits, thereby deepening our understanding of the interaction mechanisms between viruses and host cells. These findings lay the groundwork for the development of more effective antiviral drugs and vaccines. In summary, this study utilized heavy-ion irradiation-induced technology to screen for cell lines with distinct virus replication characteristics and thoroughly analyzed the functional mechanisms of the key differential gene *Cbr3*. This research not only provides a fresh perspective on the replication mechanism of the foot-and-mouth disease virus but also offers crucial theoretical foundations and practical guidance for the development of novel antiviral strategies. Future studies can build upon this work by expanding the application of ^12^C^6^ heavy-ion screening techniques, integrating more advanced biotechnologies, and delving deeper into virus-host interactions, ultimately contributing to the sustainable development of the global livestock industry.

## Supplementary Information

Below is the link to the electronic supplementary material.

Supplementary file1 (DOCX 455 KB)

Supplementary file2 (XLSX 25 KB)

## Data Availability

All the data generated or analyzed during this study are included in this published article [and its additional files].
