# Supplementary material for: The impact of 12C6 heavy ion irradiation-induced cellular mutations on the replication of the foot-and-mouth disease virus and the role of Cbr3
Source: Cell Mol Life Sci. 2025 Jun 28;82(1):261. doi: 10.1007/s00018-025-05628-6 (PMC12206219; doi:10.1007/s00018-025-05628-6)
Supplement: Supplementary file 1 — Supplementary file1 (DOCX 455 KB) [file 18_2025_5628_MOESM1_ESM.docx]

**Fig1. A**： This presents the gene ID of the *Cbr3* gene on NCBI and the CRISPR/Cas9 gene editing system. It shows the position and sequence of the sgRNA in the CDS region of the *Cbr3* gene designed in Guide design resources—Zhang Lab (squarespace.com). B: Primers are designed at 100 bp upstream and downstream of the sgRNA target site in BHK-21-KO-*Cbr3* cells. After extracting the genome of BHK-21-KO-*Cbr3* and performing PCR amplification, Sanger sequencing is employed. The sequencing results are compared with the sequence of wild-type BHK-21 cells using DNAMAN software. The result shows that BHK-21-KO-*Cbr3* has a deletion of six bases “CTTCGA” at the sgRNA site, which is marked in a red box.


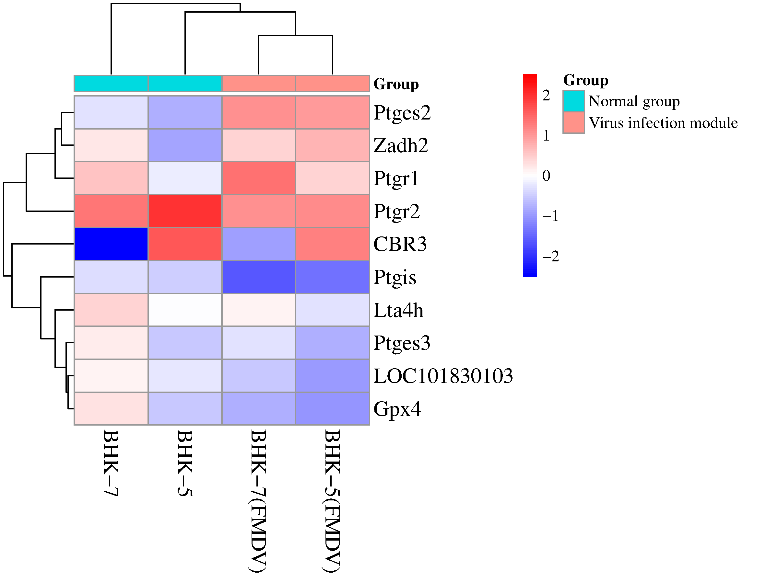


**Fig 2** ：This is the heatmap drawn for the differentially expressed proteins in the arachidonic acid metabolism pathway between BHK-5 and BHK-21 and between BHK-7 and BHK-21 before and after FMDV infection. The heatmap represents the log2 fold change of protein differences. After being standardized by z-score and clustered by complete linkage method, the heatmap is generated. The results indicate that Cbr3 shows significant up-regulation or down-regulation.


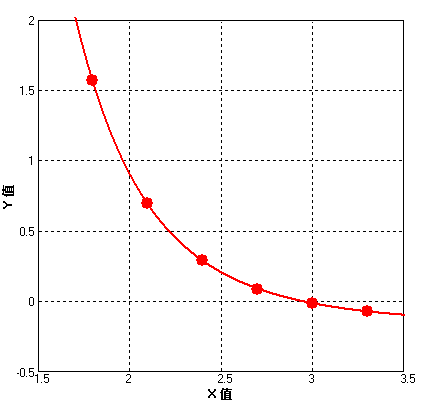


**y = (15.10469 +0.16995) / [1 + (x/ 1.18418)^4.92450^] -0.16995**

**R^2^ = 0.9999**

**Fig3**. According to the supplier (Hamster Prostaglandin E2 (PGE2) ELISA Kit, MLBio, Catalog No. ml003209), take PGE2 ELISA as the standard curve and fit a four-parameter curve.

**Fig4.** In a 96-well cell culture plate, BHK-21 cells are cultured. When the cell confluence reaches 90%, add 0.005, 0.010, 0.015, 0.020, and 0.025nmol/mL of PGE2 to the cell wells respectively. There are three replicates in each group. The blank control group is added with PBS. According to CCK-8 (CA1210, Solarbio), add the cell suspension (100 µL/well) to the 96-well plate. Place the culture plate in an incubator for pre-incubation (under the conditions of 37°C and 5% CO_2_). Add 10 µL of CCK-8 solution to each well. Incubate the culture plate in an incubator at 37°C and 5% CO_2_ for 3 hours. Measure the absorbance at 450 nm with a microplate reader. Data are presented as standard error of the mean (SEM). (∗*p* < 0.05; ∗∗*p* < 0.01; ∗∗∗*p* < 0.001).
